# Antiarrhythmic effect of the Ca^2+^-activated K^+^ (SK) channel inhibitor ICA combined with either amiodarone or dofetilide in an isolated heart model of atrial fibrillation

**DOI:** 10.1007/s00424-016-1883-9

**Published:** 2016-10-08

**Authors:** Jeppe Egedal Kirchhoff, Jonas Goldin Diness, Lea Abildgaard, Majid Sheykhzade, Morten Grunnet, Thomas Jespersen

**Affiliations:** 1 The Danish National Research Foundation Centre for Cardiac Arrhythmia, Department of Biomedical Sciences, University of Copenhagen, Blegdamsvej 3A, 2200 Copenhagen N, Denmark; 2 Department of Drug Design and Pharmacology, Faculty of Health and Medical Sciences, University of Copenhagen, Copenhagen, Denmark; 3 Acesion Pharma, Copenhagen, Denmark; 4 Lundbeck A/S, Copenhagen, Denmark

**Keywords:** Combination therapy, Antiarrhythmic drugs, QT prolongation, Amiodarone, Dofetilide, hERG1 and calcium-activated potassium channels

## Abstract

Dose is an important parameter in terms of both efficacy and adverse effects in pharmacological treatment of atrial fibrillation (AF). Both of the class III antiarrhythmics dofetilide and amiodarone have documented anti-AF effects. While dofetilide has dose-related ventricular side effects, amiodarone primarily has adverse non-cardiac effects. Pharmacological inhibition of small conductance Ca^2+^-activated K^+^ (SK) channels has recently been reported to be antiarrhythmic in a number of animal AF models. In a Langendorff model of acutely induced AF on guinea pig hearts, it was investigated whether a combination of the SK channel blocker N-(pyridin-2-yl)-4-(pyridin-2-yl)thiazol-2-amine (ICA) together with either dofetilide or amiodarone provided a synergistic effect. The duration of AF was reduced with otherwise subefficacious concentrations of either dofetilide or amiodarone when combined with ICA, also at a subefficacious concentration. At a concentration level effective as monotherapy, dofetilide produced a marked increase in the QT interval. This QT prolonging effect was absent when combined with ICA at non-efficacious monotherapy concentrations. The results thereby reveal that combination of subefficacious concentrations of an SK channel blocker and either dofetilide or amiodarone can maintain anti-AF properties, while the risk of ventricular arrhythmias is reduced.

## Introduction

The Vaughan Williams class III antiarrhythmics represent potassium-channel blockers that are often used to convert and prevent the recurrence of atrial fibrillation (AF). Amiodarone is one of the most commonly prescribed antiarrhythmic agents and is currently the most effective drug on the market for prevention of AF [26]. However, the use of amiodarone is restricted by severe non-cardiac side effects including, but not limited to, pulmonary fibrosis, photosensitivity, thyroid dysfunctions, and neurological disorders [1]. It has been reported that 18 % of patients had to discontinue the use of amiodarone because of adverse events [26]. Amiodarone is classified as a class III antiarrhythmic agent because of its inhibition of the rapid and slow delayed rectifier potassium currents I_Kr_ and I_Ks_, respectively, but it also inhibits other cardiac ion channels conducting sodium (I_Na_), calcium (I_Ca_,_L_), and potassium (transient outward— I_to_) [18]. Despite the reported prolongation of the QT interval in patients, amiodarone shows little propensity for induction of *Torsades de Pointes* (TdP) [19].

Dofetilide was, until recently, believed to be a specific I_Kr_ blocker; however, new data indicate that it also increases the late sodium current [39]. I_Kr_ is conducted through hERG1 channels, which are expressed in both atria and ventricle [15, 27]. Unlike other antiarrhythmic agents, dofetilide does not increase mortality in patients with recent myocardial infarction or congestive heart failure and is therefore important as an alternative to amiodarone for the pharmacological conversion of AF in patients at high risk of sudden cardiac death [24]. However, dofetilide prolongs the QT interval, believed to increase the risk of potentially lethal TdP arrhythmias, and has been withdrawn from the European market. The SAFIRE-D study showed an increase in AF and atrial flutter conversion rates from 9.8 to 29.9 % when increasing the dofetilide dose from 250 to 500 μg bis in die (BID) [29]. The incidences of TdP caused by dofetilide also increased in a dose-dependent manner, from 0.3 % with a dose of 250 μg BID to 10.5 % when the dose was above 500 μg BID [25].

The small conductance calcium-activated potassium (SK) channels, mediating I_SK_, are a family of potassium channel proteins with three highly homologous members (SK1, SK2, and SK3), all reportedly expressed in the heart [36], but are also known to play important functions in the CNS and vasculature [33]. SK channels, which have been reported to be localized in close proximity to L-type calcium channels, are gated following submicromolar increases in cytoplasmic calcium [38]. This suggests a unique role for these channels to translate increase in intracellular Ca^2+^ to repolarization [20]. Functionally, SK channels have been reported to play a more prominent role in the atria compared to non-diseased ventricles [36]. However, in failing human and rabbit ventricle, I_SK_ has been suggested to participate in repolarization [4, 40]. In the atria, pharmacological inhibition of these channels has been found to increase the effective refractory period (ERP) and to revert and protect against AF in a number of animal models of induced AF [7, 8, 12, 30]. Single-nucleotide polymorphisms in the KCNN3 gene encoding SK3 channels have been associated with AF risk, directly linking SK channels to clinical AF [3, 9, 10]. In isolated atrial myocytes and in trabeculae muscle, strips from patients in sinus rhythm SK channel inhibition prolonged action potential duration (APD) and ERP, suggesting the involvement of SK channel in human repolarization and the antiarrhythmic potential of SK channel inhibition [31]. In addition, we have previously reported SK inhibition to depolarize the resting membrane potential in both human and rat atria [31, 32]. Depolarization of the diastolic potential may cause voltage-dependent inactivation reducing sodium-channel availability, which will result in both slowing of the conduction and further prolongation of ERP. Several small molecule inhibitors of SK channels are known [11]. Presently, ICA seems to be the most specific, having at least 40-fold specificity over other relevant cardiac ion channels [31, 32].

We have previously reported SK channel inhibition to act synergistically with sodium channel blockers [16]; however, whether the SK channel inhibition in combination with class III antiarrhythmics has an anti-AF effect has not been addressed. Therefore, in a Langendorff AF model on guinea pig hearts, the relatively selective SK channel pore blocker, ICA, was combined with either dofetilide or amiodarone in doses that were subefficacious as a monotherapy to investigate whether it was possible to maintain efficacy and thereby reduce adverse effects.

## Methods

The studies were performed under a license from the Danish Ministry of Justice (license no. 2012/15-2934-00345) and in accordance with the Danish guidelines for animal experiments according to the European Commission Directive 86/609/EEC.

### Langendorff preparation

A total of 53 isolated guinea pig hearts were prepared as previously described [17]. Dunkin Hartley guinea pigs weighing between 350 and 500 g were anesthetized, the heart removed and mounted in a Langendorff setup (Hugo Sachs-Harvard Apparatus GmbH) and perfused through the aorta with Krebs–Henseleit solution [16] at constant oxygenation with a O_2_/CO_2_ mixture of 95/5 % at 37 °C. Monophasic action potential (MAP) electrodes were placed on the left atrium, the right atrium, the left ventricle, and the right ventricle. A pacing electrode was placed on the right atrium and the heart was allowed to stabilize for 30 min before recordings of electrophysiological parameters were initiated. The electrocardiogram (ECG) was obtained with three ECG electrodes placed in close proximity to the heart. All data were acquired using the 16-channel PowerLab system (ADInstruments). The pacing stimuli were twice the diastolic threshold with duration of 2 ms. The coronary flow was unaltered following application of compounds as compared to time-matched controls.

### Compounds

The SK blocker *N*-(pyridin-2-yl)-4-(pyridin-2-yl)thiazol-2-amine (ICA) was supplied by Acesion Pharma. All other chemicals were obtained from Sigma-Aldrich. All compounds were dissolved as stock solutions of 10 mM in DMSO. The final DMSO concentrations never exceeded 0.1 %.

### Electrophysiology

Dofetilide or amiodarone was tested alone or in combination with ICA, all in subefficacious concentrations. The atrial effective refractory period (aERP) was measured (10 basic stimuli S1 followed by a premature S2 stimulus in increasing steps of 2 ms) at baseline and every 5 min until the end of the 30-min period, with 1.0 μM dofetilide or 1.0 μM amiodarone added to the buffer either as monotherapy or in combination with 0.1 μM ICA. The aERP was measured at pacing rates of 5 and 10 Hz. Subsequently, 1 μM ACh was added to the perfusate and AF induction was attempted 40 times with burst pacing (50 Hz, 10 V) or until sustained AF (>3 min) was obtained.

In the second part of the experiments, dofetilide (3.0 μM) and amiodarone (3.0 μM) in concentrations that were effective as monotherapy were compared to the respective combination therapy on the proarrhythmic parameters: QT, triangulation, and instability of APD_90_.

### Data analysis

Poincaré plot was drawn by plotting a given APD_90_ as a function of the previous APD_90_, and short-term variability (STV) was calculated from 49 subsequent APD_90_s using (STV ¼ Σ│Dnþ1–Dn│/[49*√2]) [35].

Data analyses were performed using GraphPad Prism 5 and LabChart 7 (ADInstruments). Data are summarized using the mean ± SEM. Repeated measures two-way ANOVA with Bonferroni post hoc tests was used to compare mean values of aERP, QT, and triangulation. For the statistics, the AF durations were log-transformed and repeated measures one-way ANOVA with Bonferroni post hoc tests was used to compare the mean values of the AF duration, ΔQT, and STV. Values of *P <* 0.05 were considered statistically significant (denoted by asterisks in figures). Values of *P <* 0.01 and *P <* 0.001 are denoted by ** and ***, respectively.

## Results

**Fig. 1:**
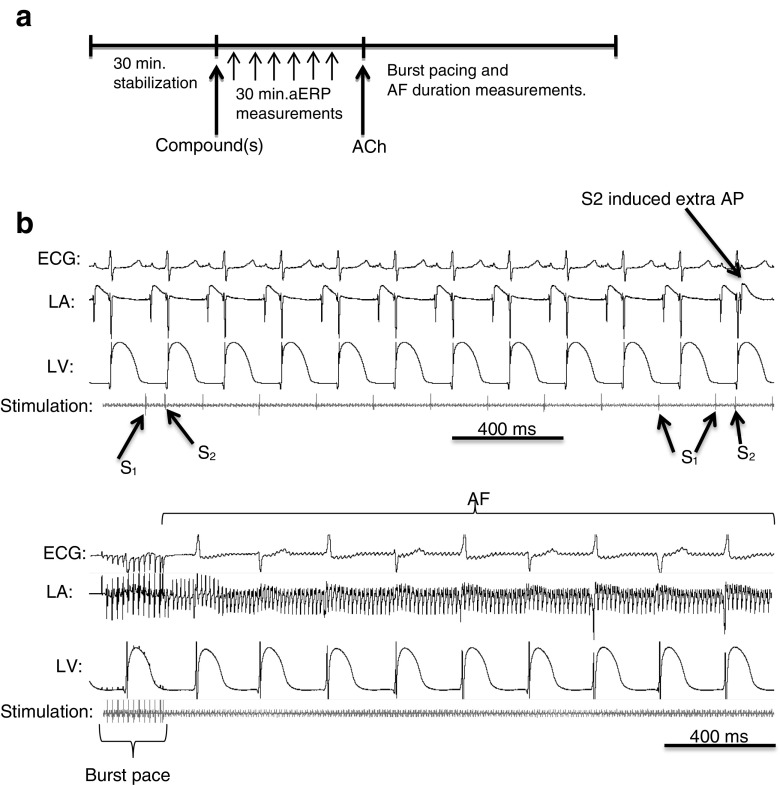
Induction and treatment protocol together with representative control recordings. **a** Experimental protocol for aERP and AF duration measurements. After 30 min of stabilization following mounting in the Langendorff configuration, compound or combination of compounds was added and aERP was measured every 5 min for 30 min. The hearts were paced at 5 Hz, except while performing the 10-Hz aERP measurements. Induction of AF was the attempted by shortening of ERP by ACh and burst pacing. **b** Representative traces from a control experiment displaying the procedure for measuring aERP (*top*) and induction of persistent AF (*bottom*). LA left atrium, LV left ventricle

Guinea pig hearts express a number of the same ionic currents as humans, including I_Kr_ and I_Ks_ [2], and both atrial and ventricular arrhythmias can be triggered in these hearts [6, 16]. Therefore, to investigate whether a given pharmacological treatment could revert and prevent acutely induced AF, we isolated guinea pig hearts and mounted them in a Langendorff configuration where they were perfused with oxygenated 37 °C saline buffer. In this model, test compounds can be supplied to the buffer, electrical stimulation can be applied (used both for pacing and refractory measurements), and electrical activity recorded by monophasic action potential (MAP) and ECG electrodes. Further, application of acetylcholine (ACh) drastically increases AF vulnerability by reducing the refractoriness of the atrial tissue and if burst pacing is applied, AF can be induced (Fig. 1) [17].

Before performing the combination studies with ICA together with either dofetilide or amiodarone, the effective dose for reverting AF, when supplied as monotherapy, was tested. These investigations showed that 0.3 μM ICA, 3.0 μM dofetilide, and 3.0 μM amiodarone effectively reverted AF (Table 1). As a consequence, 0.1 μM ICA, 1 μM dofetilide, and 1 μM amiodarone were applied in the combination study.

**Table 1:** Data from the experiments: Displaying atrial effective refractory periods (aERP), AF durations, the difference in QT interval at 5 Hz before and after addition of compounds listed as ΔQT

	Number	aERP 5 Hz (ms)	aERP 10 Hz (ms)	AF duration (s)	ΔQT (ms)
Control	6	56 ± 2	48 ± 2	35.8 ± 7.4	−2.6 ± 1.2
ICA (0.1 μM)	7	68 ± 3	58 ± 3	34.7 ± 8.0	1.4 ± 2.7
Dofetilide (1.0 μM)	7	89 ± 2^a,b^	84 ± 3^a,b^	36.2 ± 4.5	11.7 ± 4.6
ICA (0.1 μM) and dofetilide (1.0 μM)	6	94 ± 4^a,b^	91 ± 4^a,b^	1.1 ± 0.9^a,b,c^	7.4 ± 3.4
Amiodarone (1.0 μM)	6	66 ± 4	67 ± 5^a^	25.9 ± 6.7	5.8 ± 4.6
ICA (0.1 μM) and amiodarone (1.0 μM)	8	86 ± 4^a,b,c^	90 ± 3^a,b,c^	8.0 ± 4.4^a,b,c^	4.6 ± 2.2
Dofetilide (3.0 μM)	7	ND	ND	0.4 ± 0.1	31.1 ± 5.9 ^a,b,e,d^
Amiodarone (3.0 μM)	6	ND	ND	1.2 ± 0.8^a^	5.3 ± 4.9

The aERP values noted is after 30 min of compound perfusion

*ND* not done

^a^Significant difference from control

^b^Significant difference from ICA (0.1 μM )

^c^Significant difference from amiodarone (1.0 μM)

^d^Significant difference from dofetilide (1.0 μM)

^e^Significant difference from ICA (0.1 μM) and dofetilide (1.0 μM)

### Atrial refractoriness with combination therapy

**Fig. 2:**
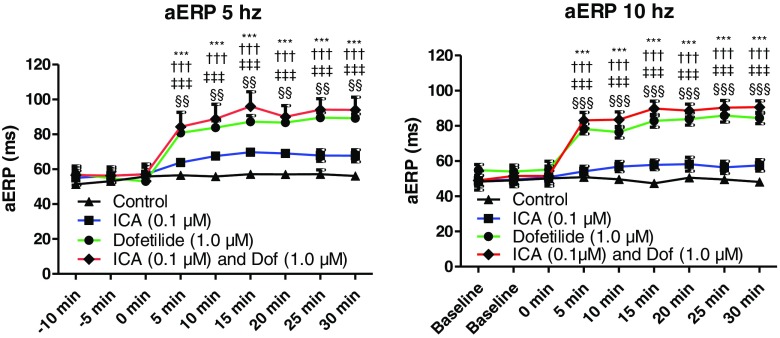
Atrial refractoriness following perfusion with dofetilide and ICA. Atrial effective refractory period (*aERP*) of ICA and dofetilide (*Dof*) in concentrations subefficacious as independent monotherapies, either alone or in combination, at pacing rates of 5 and 10 Hz. *ICA and dofetilide significant difference from the control. †ICA and dofetilide significant difference from ICA. ‡Dofetilide significant difference from control. §Dofetilide significant difference from ICA

To investigate whether the combinations of ICA and either dofetilide or amiodarone have additive effects on atrial electrophysiology and AF prevention, a series of Langendorff experiments was performed (Table 1). aERPs were measured every 5 min prior to and after compound perfusion (Fig. 1). The SK blocker ICA, at a concentration of 0.1 μM, only revealed a non-significant tendency toward increase in aERP at both 5 and 10 Hz compared to control (Fig. 2 and Table 1). Both 1.0 μM dofetilide alone and the combination of dofetilide and ICA increased aERP significantly at both 5 and 10 Hz compared to control and ICA alone, but no differences in aERP were observed between these two treatment groups (Fig. 2 and Table 1).

**Fig. 3:**
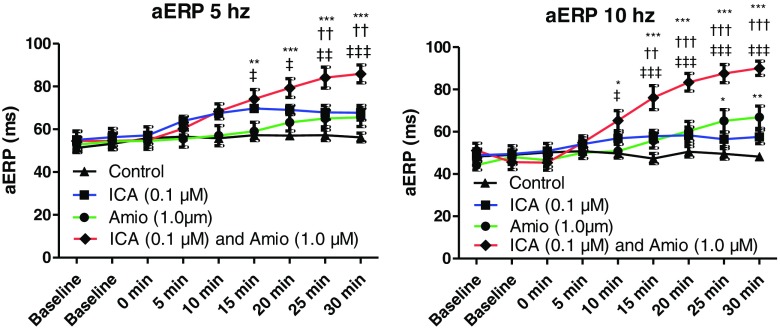
Atrial refractoriness following perfusion with amiodarone and ICA. Atrial effective refractory period (*aERP*) of ICA and amiodarone (*Amio*) at concentrations subefficacious as independent monotherapies, either alone or in combination, at pacing rates of 5 and 10 Hz. *Significant difference from the control. †Significant difference from ICA. ‡Significant difference from amiodarone

Perfusion with 1.0 μM amiodarone increased aERP, although only significantly at 10 Hz, compared to control. This effect of borderline aERP prolongation observed for both ICA and amiodarone is drastically amplified when combining the two compounds (Fig. 3 and Table 1). The onset of effect on aERP was slower for the ICA + amiodarone group than for the dofetilide and ICA + dofetilide group, indicating that amiodarone needs longer time to reach the ion channel targets (I_Kr_ and I_Ks_) than does dofetilide.

### AF prevention with combination therapy

**Fig. 4:**
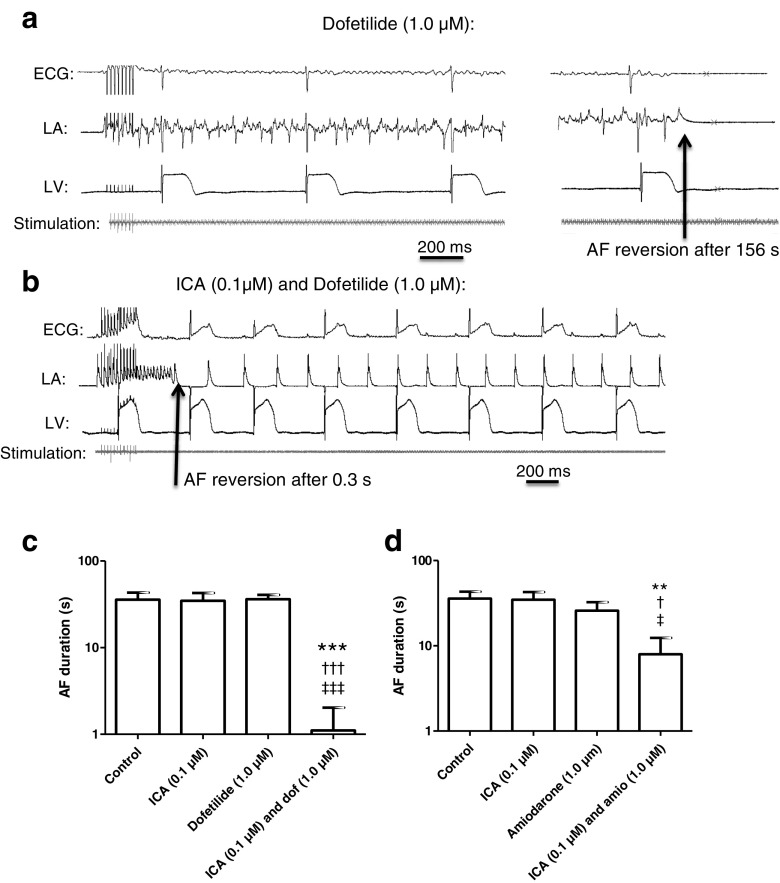
Combination treatment with either dofetilide or amiodarone and ICA reduces AF burden. **a** Representative recordings from the induction of AF after 30 min of application of dofetilide (1.0 μM) (start and reversion of AF shown, while the middle 154 s is not shown). AF was induced by addition of acetylcholine and burst pacing. *LA* Left atrium, *LV* left ventricle. **b** Combination of ICA (0.1 μM) and dofetilide (1.0 μM). Note, the addition of ACh induces third degree AV block in guinea pig Langendorff hearts and ventricles therefore following their own intrinsic beating. **c** AF duration for control, ICA, dofetilide, and ICA + dofetilide groups. The combination with ICA and dofetilide reduced AF duration compared to the control and the monotherapies. The monotherapies did not reduce AF durations compared to the control deeming them subefficacious. **d** The combination of ICA and amiodarone reduced the AF durations compared to control and the subefficacious monotherapies. *Significant difference from the control. †Significant difference from ICA. ‡Significant difference from dofetilide. §Significant difference from amiodarone

With the test compound still in the perfusion buffer, 1.0 μM ACh was added and burst pacing induction of AF was attempted 40 times. In non-protected hearts, this resulted in short runs of AF with an average of 35 s (representative recording in Fig. 4a). In protected hearts, this AF duration was significantly reduced or even absent (representative recording in Fig. 4b). The AF duration time was measured in the time-matched control hearts, in hearts receiving monotherapy, and in hearts with either combination of drugs. The monotherapies of ICA (0.1 μM) and dofetilide (1.0 μM) did not reduce the AF durations compared to the control (Table 1). In contrast, with 3.0 μM dofetilide AF durations were reduced compared to the control (Table 1). The combination therapy of 0.1 μM ICA and 1.0 μM dofetilide significantly reduced the average AF duration compared to the control, ICA, and 1.0 μM dofetilide alone (Fig. 4c). 1.0 μM amiodarone as a monotherapy did not reduce the AF duration significantly compared to the control (Table 1). However, increasing the concentration to 3.0 μM amiodarone resulted in reduced AF duration (Table 1). When 0.1 μM ICA and 1.0 μM amiodarone were combined, the AF duration was significantly reduced compared to the control, ICA, and 1.0 μM amiodarone alone (Fig. 4d).

### Proarrhythmic effects on ventricular electrophysiology

**Fig. 5:**
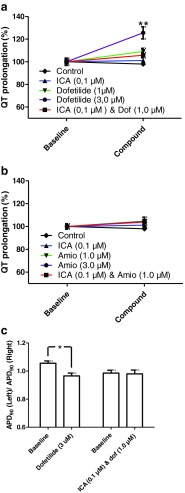
Compound-induced effect on QT interval. Potential QT interval prolongation caused by the compounds were investigated by comparing relative QT durations before and after 30-min perfusion with compounds. **a** Investigations of the dofetilide groups revealed that only dofetilide (3.0 μM) increased the QT interval significant compared to the combination of ICA (0.1 μM) and dofetilide (1.0 μM), which were the only groups that proved antiarrhythmic. **b** In the amiodarone groups, no QT interval prolongation was found. **dofetilide (3.0 μM) significant difference from the combination of ICA (0.1 μM) and dofetilide (1.0 μM). **c** Spatial dispersion in repolarization between right and left ventricle recorded before and after perfusion with dofetilide (3.0 μM) and dofetilide (1.0 μM) and ICA (0.1 μM). The repolarization time was recorded at 90 % repolarization (APD90) with MAP electrodes placed on the epicardial surface of the right and left ventricles

For some pharmacological compounds, a correlation between QT prolongation and increased propensity for TdP has been demonstrated. We therefore investigated the QT prolonging effect of the dose of dofetilide and amiodarone that was effective in preventing AF as a monotherapy and compared this to the respective combinations in concentrations that were effective in combination but subefficacious as monotherapies. Dofetilide (3.0 μM) increased the QT interval by 26 % compared to baseline (Fig. 5), which was significantly higher compared to the 6 % prolongation caused by the combination of ICA (0.1 μM) and dofetilide (1.0 μM). The 3.0 μM amiodarone did not increase the QT interval compared to the baseline or the combination of ICA (0.1 μM) and amiodarone (1.0 μM).

To investigate the spatial dispersion in repolarization, APD_90_ recorded from the left and right ventricle was analyzed (Fig. 5). Following perfusion with 3.0 μM dofetilide, both right and left ventricles have prolonged repolarization time. However, the right ventricular APD_90_ was increased more profoundly than the left ventricular APD_90_, indicating an increased spatial dispersion in repolarization. Perfusion with lower concentration of dofetilide (1.0 μM) together with 0.1 μM ICA did not increase the spatial dispersion.

**Fig. 6:**
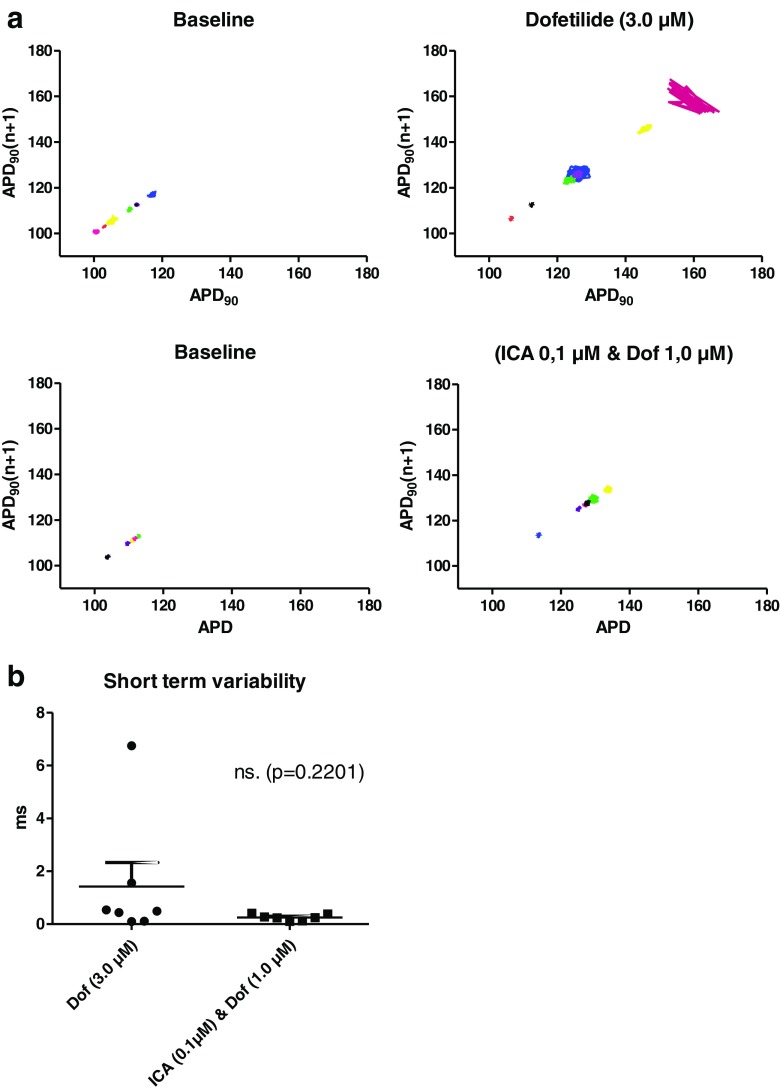

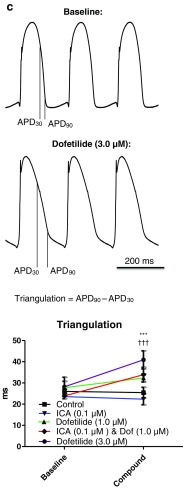
Dofetilide induced effects on ventricular repolarization. **a** Poincaré plots generated by plotting one APD_90_ against the next to illustrate beat-to-beat instability in repolarization. Instability of dofetilide in a concentration of 3.0 μM is observed in two of the experiments by increased complexity of the plot. No instability was observed with the combination of ICA (0.1 μM) and dofetilide (1.0 μM). In the Poincaré plot, each *color* represents one experiment (seven in total in each group) at baseline and during drug treatment. **b** To quantify the observations in the Poincaré plots, short-term variability in repolarization was calculated. These analyses confirm two of the 3.0 μM hearts having a large variability. **c** Representative traces displaying triangulation of the action potential by dofetilide (3.0 μM). Triangulation, which is a measure for the time in phase 3 repolarization, was calculated by APD_90_-APD_30_. ***significant difference between the control and dofetilide (3.0 μM). †††significant difference from ICA (0.1 μM)

Another important parameter for estimating potential ventricular proarrhythmicity is the variability in the repolarization of the action potentials. This was investigated by Poincaré plots where one APD_90_ value is plotted as a function of the preceding APD_90_ value (Fig. 6a, b). Application of 3.0 μM dofetilide resulted in a larger dispersion in variability for some of the hearts while for the combination group with 0.1 μM ICA and 1.0 μM dofetilide, no major increase in variability was observed. This was quantified by calculating short-term variability (STV) showing a part of the hearts with increased beat-to-beat dispersion in repolarization. However, although two of the six hearts had a profound increase in STV, the mean STV was not statistically significant (Fig. 6c).

Finally, an increased triangulation, calculated as the time period between APD_30_ and APD_90_, has been suggested to be a measure of proarrhythmicity [13]. This time period has been termed the vulnerable window, where there is a risk of experiencing early after-depolarizations, which may develop into arrhythmia [14]. Dofetilide has previously been reported to increase triangulation [23], and, indeed, 3.0 μM dofetilide was calculated to increase triangulation in these guinea pig hearts compared to the control and ICA (0.1 μM). Both 1.0 μM dofetilide alone and together with 0.1 μM ICA revealed a trend toward increased triangulation compared to the control.

## Discussion

Combination therapy has the potential to reduce the dose of class III antiarrhythmics needed while maintaining their anti-AF efficacy. Amiodarone is among the most commonly prescribed antiarrhythmics, but its use is limited by severe dose-related non-cardiac side effects. Dofetilide improves atrial fibrillation and atrial flutter conversion rates, but with increasing doses grows the risk of TdP.

The SK channel is a novel, not yet clinically tested, target for treatment of AF. Pharmacological inhibition of these channels increases aERP in both human and experimental models and protects against AF in animal models [8, 30, 31]. SK channels are highly interesting in an AF perspective, not only because SK channels in the heart primarily seem to be of functional importance in the atria but also due to the fact that SK channels are gated by submicromolar intracellular calcium concentrations. This gives SK channels a potential pivotal role in sustaining AF, in particular in newly developed AF, where intracellular calcium handling is compromised [37]. An increased I_SK_ during AF would be expected to have a profound effect on the repolarization leading to shorter action potentials and decreased refractoriness [34], which would be expected to be a substrate for the maintenance of AF [21]. However, whether this is the mechanism making SK channel blockers antiarrhythmic in several different models of AF still remains to be investigated.

Prolongation of atrial effective refractory period is believed to be an important electrophysiological principle for treating AF [5]. At a subefficacious concentration in monotherapy, dofetilide increased aERP to a similar extent as the combination of ICA and dofetilide. However, only the combination of ICA and dofetilide significantly reduced the AF duration. This discrepancy shows that aERP measurements during sinus rhythm are not the only pivotal parameter in evaluating the antiarrhythmic potential of a given compound. A potential explanation for the additive effect by ICA is that, during AF, the intracellular diastolic Ca^2+^ concentration rises [22], which may lead to an increased activation of SK channels [34]. An increased I_SK_ during AF would have an effect on repolarization in both the diastolic interval and during the action potential. As the diastolic interval is profoundly depolarized to potentials around −65 mV during AF, the majority of the cardiac sodium channels will be inactivated [28, 32], which will lead to reduced upstroke velocity of the action potential and reduced conduction velocity. With an increased I_SK_ during AF, a fraction of the K^+^ conductance in the diastolic interval will be originating from the SK channels. As a consequence, pharmacological blockage of these SK channels could, during AF, reduce the repolarization force throughout the depolarization, which could provide longer action potentials, and thereby prolong the time before reaching the point where an adequate fraction of sodium channels are released from voltage- and time-dependent inactivation to support a new action potential upstroke. Hence, an increased I_SK_ during AF action potentials would be expected to reduce both APD and ERP, whereby pharmacological inhibition of SK channels could potentially prolong refractoriness. This is expected to be antiarrhythmic. Based on this, we suggest that pharmacological blockage of SK channels has a more profound effect during AF, which would explain the relatively small electrophysiological changes recorded following SK channel block of atrial tissue in sinus rhythm.

Dofetilide has predominantly been reported to affect the refractoriness of the atria through the hERG1 block. The hERG1 channels underlie the rapid delayed rectifier K^+^ current I_Kr_, which plays an important role in phase 3 repolarization. As the combination of ICA and dofetilide drastically reduces the AF duration as compared to the respective monotherapies, our results indicate a synergistic effect by combining these two compounds having different molecular targets.

The prolongation of QT and subsequent risk of TdP caused by dofetilide has been well established in the clinic [25]. At a concentration that was effective in the prevention of AF as a monotherapy, dofetilide produced a marked increase in the QT interval in isolated guinea pig hearts. When subefficacious monotherapy concentrations of ICA and dofetilide were combined, it not only showed an anti-AF effect, but in addition, the combination did not increase the QT interval significantly. Another important parameter in investigating potential proarrhythmic ventricular side effects is dispersion in repolarization. When hearts where treated with an effective dose of dofetilide, some of the hearts showed a clear increase in the beat-to-beat repolarization time. In addition, the spatial dispersion in repolarization between the right and left ventricle was found increased with 3.0 μM dofetilide. Neither increase in temporal nor in spatial dispersion was observed in the combination group of ICA and dofetilide. Furthermore, triangulation of the ventricular action potential has also been reported as a critical parameter for estimating proarrhythmicity [14]. At 3.0 μM, dofetilide significantly increased triangulation. Reducing the dose to 1.0 μM, both with and without ICA decreased the degree of triangulation, confirming that the dose of dofetilide is important in making the action potential more triangular. The results thereby show that the combination of an SK channel blocker and dofetilide applied in lower concentrations may maintain antiarrhythmic efficacy and, at the same time, reduce the risk of TdP and other ventricular arrhythmias.

Amiodarone is one of the most commonly used anti-AF drugs. Chronic treatment of amiodarone is often restricted by non-cardiac dose-dependent adverse effects [41]. It is therefore of great interest to investigate whether the effective concentration of amiodarone can be lowered by combining it with another antiarrhythmic compound. Amiodarone (1.0 μM) and 0.1 μM ICA applied as monotherapies both gave borderline prolongation of ERP. When combining the compounds, a drastic ERP prolongation was found. This additive effect was confirmed when analyzing AF duration where the monotherapies were ineffective in shortening AF duration, and the combination of ICA and amiodarone gave profound shortening. To investigate potential ventricular adverse effects of the compounds, the QT intervals were analyzed. Amiodarone did not increase the QT interval at either a high concentration or lower concentration in combination with ICA. Our results thereby reveal that by combining an SK blocker with the multichannel blocker amiodarone, a lower dose of amiodarone is needed to reduce AF duration.

### Study limitations

In the ACh and burst pacing Langendorff model, AF is induced acutely and the impact of structural remodeling, known to be present in more persistent forms of AF, is therefore not accounted for. Hence, the investigations performed do not fully reflect the conditions observed in the clinic and extrapolation of data should, therefore, be performed with caution. For example, in long-lasting human AF, the expression of SK channels has been reported to be reduced [31]. Treatment of patients with longer periods of AF with SK channel blockers may therefore be challenging.

## Conclusion

The combination of ICA with dofetilide or amiodarone in concentrations ineffective as independent monotherapies reduced the AF durations compared to both control and monotherapies. At a concentration that was effective as a monotherapy, dofetilide produced a prolongation of the QT interval that was not observed with effective doses of ICA and dofetilide in combination.

Hence, the combination of subefficacious concentrations of an SK channel blocker and either dofetilide or amiodarone could possibly maintain the anti-AF properties, whereby the risk of ventricular arrhythmias and non-cardiac side effects will be reduced. Although these observations suggest potential new treatment modalities for AF, a thorough understanding of the pathophysiology, optimal pharmacokinetics, and potential drug–drug interactions is a prerequisite to determine whether the observed findings can translate into the human in vivo situation.

## Abbreviations

AF: Atrial fibrillation
ICA: N-(pyridin-2-yl)-4-(pyridin-2-yl)thiazol-2-amine
SK: Small conductance Ca^2+^-activated K^+^
ACh: Acetylcholine
TdP: *Torsade de Pointes*
BID: Bis in die (twice a day)
